# New Books

**Published:** 2009-10

**Airborne Radioactive Contamination in Inhabited Areas**

K.G. Andersson

St. Louis, MO:Elsevier, 2009. 250 pp. ISBN: 978-0-08-044989-0, $155

**Air Pollution**

Jes Fenger, C. Jens Tjell, eds.

New York:Springer, 2009. 488 pp. ISBN: 978-1-84755-865-7, $189

**Air Quality and Ecological Impacts: Relating Sources to Effects**

Allan H. Legge

St. Louis, MO:Elsevier, 2009. 334 pp. ISBN: 978-0-08-095201-7, $185

**ASEAN Environmental Law, Policy and Governance**

Kheng-Lian Koh, ed.

Hackensack, NJ:World Scientific, 2009. 736 pp. ISBN: 978-981-4261-18-0, $120

**Averting Disaster: Science for Peace in a Perilous Age**

William A Barletta, Henning Wegene

Hackensack, NJ:World Scientific, 2009. 212 pp. ISBN: 978-981-4289-40-5, $48

**Bioinformatics Research and Applications**

Ion Mandoiu, Giri Narasimhan, Yanqing Zhang, eds.

New York:Springer, 2009. 336 pp. ISBN: 978-3-642-01550-2, $79.95

**Climate Change: Observed Impacts on Planet Earth**

Trevor Letcher, ed.

St. Louis, MO:Elsevier, 2009. 492 pp. ISBN: 978-0-444-53301-2, $90

**Doing Science: Design, Analysis, and Communication of Scientific Research**

Ivan Valleta

New York:Oxford University Press, 2009. 352 pp. ISBN: 978-0-19-538573-1, $34.95

**Environmental Justice and Sustainability in the Former Soviet Union**

Julian Agyeman, Yelena Ogneva-Himmelberger, eds.

Cambridge, MA:MIT Press, 2009. 320 pp. ISBN: 978-0-262-51233-6, $25

**Facing Global Environmental Change**

H.G. Brauch, Ú. Oswald Spring, J. Grin, C. Mesjasz, P. Kameri-Mbote, N.C. Behera, B. Chourou, H. Krummenacher, eds.

New York:Springer, 2009. 1,588 pp. ISBN: 978-3-540-68487-9, $379

**Information Resources in Toxicology**

Philip Wexler, P.J. Hakkinen, Asish Mohapatra, Steven G. Gilbert

St. Louis, MO:Elsevier, 2009. 1344 pp. ISBN: 978-0-12-373593-5, $199.95

**International Seminar on Nuclear War and Planetary Emergencies**

R. Ragaini, ed.

Hackensack, NJ:World Scientific, 2009. 800 pp. ISBN: 978-981-4289-12-2, $198

**Security and Environmental Change**

Simon Dalby

Hoboken, NJ:John Wiley & Sons, Inc., 2009. 200 pp. ISBN: 978-0-7456-4291-8, $64.95

**The Rise of China and India**

Lam Peng Er, Lim Tai Wei, eds.

Hackensack, NJ:World Scientific, 2009. 176 pp. ISBN: 978-981-4280-33-4, $80

**Transportation in a Climate-Constrained World**

Andreas Schäfer, John B. Heywood, Henry D. Jacoby, Ian A. Waitz

Cambridge, MA:MIT Press, 2009. 384 pp. ISBN: 978-0-262-51234-3, $27

